# Project Energize: intervention development and 10 years of progress in preventing childhood obesity

**DOI:** 10.1186/s13104-016-1849-1

**Published:** 2016-01-26

**Authors:** Elaine Rush, Carolyn Cairncross, Margaret Hinepo Williams, Marilyn Tseng, Tara Coppinger, Steph McLennan, Kasha Latimer

**Affiliations:** Child Health Research, Auckland University of Technology, Auckland, New Zealand; Kinesiology Department and Center for Solutions Through Research in Diet and Exercise (STRIDE), California Polytechnic University, San Luis Obispo, California USA; Department of Sport, Leisure & Childhood Studies, Cork Institute of Technology, Cork, Ireland; Sport Waikato, Hamilton, New Zealand

**Keywords:** Children, Māori, Indigenous, Physical activity and nutrition, Healthy weight gain

## Abstract

Prevention of childhood obesity is a global priority. The school setting offers access to large numbers of children and the ability to provide supportive environments for quality physical activity and nutrition. This article describes Project Energize, a through-school physical activity and nutrition programme that celebrated its 10-year anniversary in 2015 so that it might serve as a model for similar practices, initiatives and policies elsewhere. The programme was envisaged and financed by the Waikato District Health Board of New Zealand in 2004 and delivered by Sport Waikato to 124 primary schools as a randomised controlled trial from 2005 to 2006. The programme has since expanded to include all 242 primary schools in the Waikato region and 70 schools in other regions, including 53,000 children. Ongoing evaluation and development of Project Energize has shown it to be sustainable (ongoing for >10 years), both effective (lower obesity, higher physical fitness) and cost effective (one health related cost quality adjusted life year between $18,000 and $30,000) and efficient ($45/child/year) as a childhood ‘health’ programme. The programme’s unique community-based approach is inclusive of all children, serving a population that is 42 % Māori, the indigenous people of New Zealand. While the original nine healthy eating and seven quality physical activity goals have not changed, the delivery and assessment processes has been refined and the health service adapted over the 10 years of the programme existence, as well as adapted over time to other settings including early childhood education and schools in Cork in Ireland. Evaluation and research associated with the programme delivery and outcomes are ongoing. The dissemination of findings to politicians and collaboration with other service providers are both regarded as priorities.

## Background

Over the last decade, the increasing prevalence of obesity and overweight has prompted calls for global action [1] and a global action plan [2]. A recent series in the Lancet [3] addresses the need for global action to impose restrictions on advertising energy-dense and nutrient-poor foods to children, taxes on sugary drinks and regulation of food nutritional quality and availability in schools. A 2011 Cochrane review [4] of interventions for prevention of obesity in children concluded that there was strong evidence of beneficial effects for child obesity programmes on BMI, particularly for children aged 6–12 years. Five of the six promising policies and strategies were centered on schools and the sixth strategy was around parental support and home activities. A recent meta-analysis of school based programmes [5] found that longer interventions (>1 year) were more effective than shorter ones.

While randomised controlled trials are considered the best level of evidence for effectiveness, very little is known about efficacy when interventions are translated into real world settings [6]. One of the challenges is that few trials last more than a year; therefore, programme sustainability and ability to maintain effects of the intervention are not known. Feasibility of translation into other settings is also an important research question. In this article, we describe Project Energize, a school-based nutrition and physical activity (PA) programme initiated in the Waikato region of New Zealand in 2003, and key features contributing to its expansion and maintenance. The objective is to describe a programme that has been successful in terms of sustainability and growth (including community and international partnerships), so that others might replicate and adopt similar practices, initiatives and policies.

## Project Energize

### Protocol

In 2003, the Waikato District Health Board recognised that there was a need to improve the health and wellness of children through co-ordinating a comprehensive, adequately resourced and sustainable Healthy Eating—Healthy Living programme that would have long-term benefits. The response was the establishment of Project Energize, a through-school nutrition and PA programme in the Waikato region, delivered by Sport Waikato. Sport Waikato is one of 14 not for profit, regional sports trust in New Zealand who sit under the umbrella of Sport NZ and work with their communities to get and keep all people physically active and healthy for life. Funding for regional sport trusts come from Sport NZ, government, health providers, funding partners, private organisations and bequests.

The Project Energize programme aims to improve the overall health through increased physical activity and healthier eating and thus reduce the rate of weight gain of Waikato primary school children; of whom 34 % are Māori [7] and the indigenous people of New Zealand (Aotearoa).

Project Energize began as a quasi-experimental randomized by school cluster, controlled trial of 62 programme schools (including 11,090 children) and 62 matched control schools (10,780 children) between 2004 and 2006 [8]. Following a predetermined randomisation process the 124 schools involved were drawn from 139 who were approached (method paper). Underlying the programme are nine healthy eating goals and seven PA goals that have remained in place since the inception of the programme (Table 1). Wording has changed slightly to incorporate the adaptations made to various national initiatives, such as the National Heart Foundation School Food Programme and Active Schools campaign. The programme is aligned with government food, nutrition and PA guidelines [9, 10] and therefore works alongside and with other agencies such as the Heart Foundation. All messages and activities delivered by oral, written and demonstration modes through the programme are composed in ways to be clear, concise and consistent across all intervention components and can be mapped to the programme goals.

**Table 1:** Project Energize healthy eating and physical activity goals

Healthy eating
1. To encourage and promote water as the best drink
2. To ensure water is available in class
3. To encourage the consumption of milk and other high calcium foods every day
4. To encourage an increase in fruit and vegetable consumption
5. To advocate for and encourage improvement in food brought from home especially a reduction in the amount of high energy/low nutrient food
6. To encourage and advocate for an increase in availability of healthy choices at school and a decrease in availability of high energy/low nutrient foods
7. To increase the awareness of the importance of breakfast and encourage a breakfast habit
8. To work towards registration in the National Heart Foundation School Food Programme
9. To work towards consistent nutrition messages in all aspects of school and community interaction e.g., healthy fundraising options
Physical activity
1. To encourage a minimum of 20 min quality daily physical activity
2. To advocate for and encourage lunchtime physical activity at least twice a week
3. To encourage and advocate for at least 5 min of “home play” every day
4. To encourage a reduction in sedentary time especially screen time if over 2 h a day
5. To raise awareness of incidental activity opportunities at home and school
6. To raise awareness of the importance of children learning fundamental movement skills and movement literacy
7. To encourage and advocate for links to “Active Schools” and the philosophy of active schools

The process of engagement with schools followed a sequential pattern of invitations and meetings to present the vision and goals of the programme to the community. At each school a face to face introduction of the programme is provided to school leaders to explain what the programme would mean to them—e.g., one Energizer looking after 6–12 schools in close geographical location and a standard memorandum of understanding signed by the school and a representative of Project Energize. This memorandum explicitly states expectations of who, what and when activities would happen. A lead teacher is appointed as a point of contact for the assigned Energizer. With the Energizer the teacher undertakes a school stocktake detailing what policies, guidelines and practices are currently in place in their school. The current environment for healthy eating and PA is also assessed. From this information, the needs of the school are derived and ranked according to what the school and the Energizer are realistically able to undertake to do in the next year to work towards the goals of the programme. This results in a tailored action plan that is agreed on by the Energizer and the school for one academic year. For example one school may wish to focus on teaching fundamental movement skills (PA goal 5) and another on the quality of food sold at the school canteen (students are not provided with food and often bring lunch from home) and gaining a healthy heart award—healthy eating goal (HE goal 8).

Moreover, the mode of delivery, through a school-specific ‘Energizer’ (a trained PA and nutrition change agent), means that each school receives, in addition to “usual business”, a unique, externally-funded service tailored to the needs of that school. The Energizer fields all school interactions, programmes and activities to do with nutrition and PA and is effectively the school go-to-person to ensure that nutrition and PA actions, policies and changes to practice within schools are aligned with governmental goals and are time efficient—for example, delivery of the Kiwi Swim Safe programme by Swimming NZ to a cluster of schools rather than one by one.

### Evaluation

Measurements of outcomes related to health, knowledge and behaviours of children, parent and staff was conducted according to the guidelines laid down in the Declaration of Helsinki and approved by the Waikato (now Northern Y) Ethics Committee. Both the usual caregiver and the child provided signed informed consent before any measurements were made. The 2004–2006 trial was registered in the Australasian Clinical Trials Registry ACTRN12610000132044.

Findings from the 2004–2006 randomized, by cluster, controlled trial have been published previously and showed evidence with follow-up measures (by external staff) of children (686 boys and 662 girls) aged 5 (1926) and 10 (1426) years (692 interventions and 660 controls) of a reduced accumulation of body fat in younger children, a reduced rate of rise in systolic blood pressure in older children [11], and in a substudy lower vitamin D insufficiency [12] compared with children in control schools. Further, a follow-up evaluation in 2011 of health and fitness outcomes was conducted by Energizers over a six-week period with 2474 7 year olds and 2330 10 year olds from 193 of the 243 schools showed improved BMI and run speed outcomes [13] for Energized children compared to historical controls. Data on BMI change measured in the 2011 evaluation [14] were also used as the basis for a lifetime cost effectiveness analysis [15], which concluded that the programme, at $NZ45/child/year, was indeed cost-effective from the health treatment payer’s perspective, and “would improve quality and length of life when compared with other obesity prevention programmes previously assessed with the same model.” Regular audits of Sport Waikato’s spending by the Waikato District Health Board show that a large proportion (>90 %) of the money is spent on the Energizers who are in-the-field and in schools the majority of any school day (personal communication, Sport Waikato management).

## Expansion to other schools in NZ

**Fig. 1:**
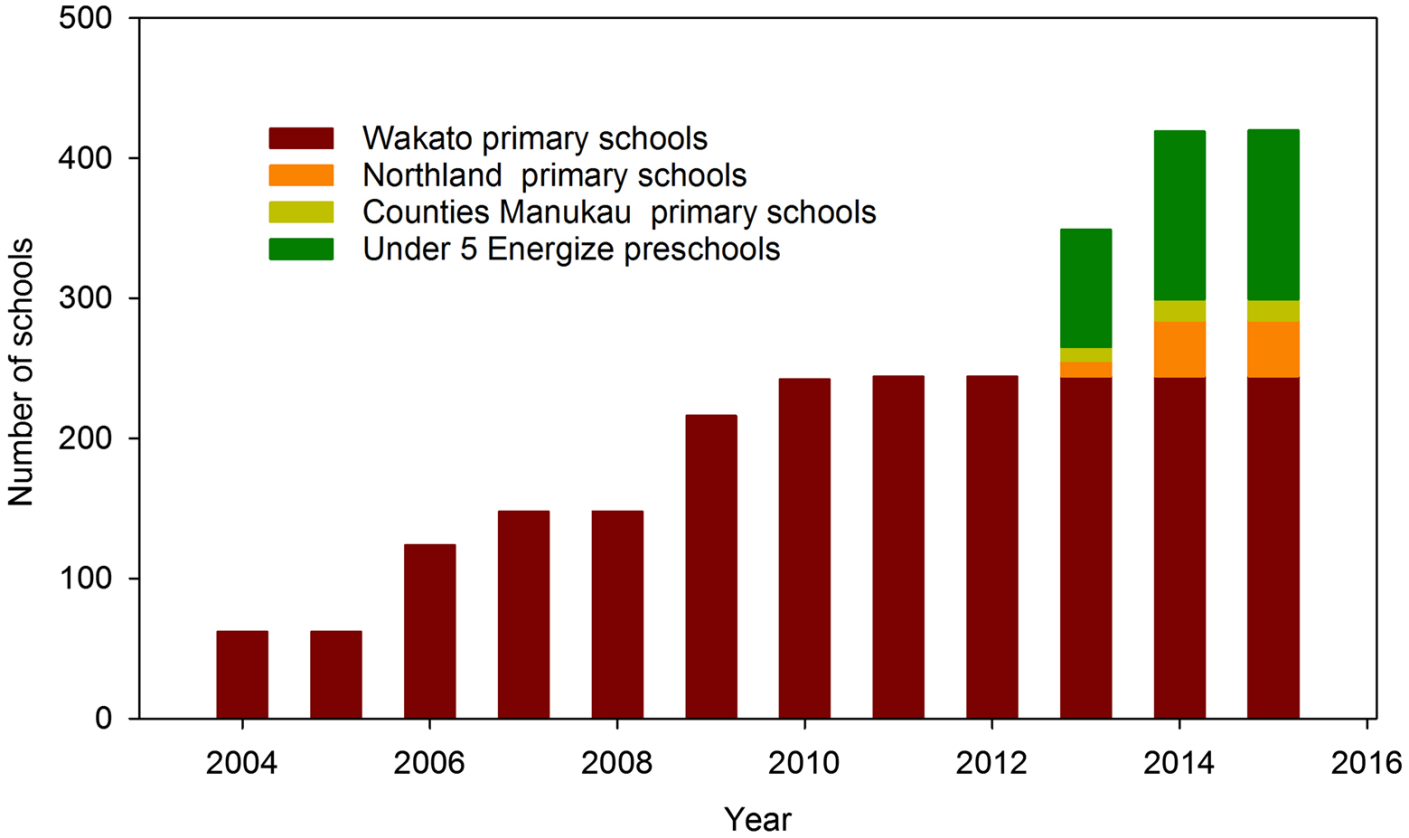
Progression of engagement of the Project Energize programme with schools

In 2004, the first 124 schools were approached about the randomized trial, and 62 were randomized to receive the intervention. Since then, the number of schools who have signed up to the programme has increased (Fig. 1). By 2012, all 242 and 44,000 children in Waikato primary schools including special needs schools, were engaged in the initiative, served by 27 Energizers employed by Sport Waikato. Subsequently, in 2013, schools from two other regions in NZ began to receive the Energize programme, with mentorship from the Waikato Energizers and using the resources developed by Sport Waikato (Table 2). In each region, the programme is delivered by the regional sports trust (Sport Waikato, Counties Manukau Sport, Sport Northland) and funded by the sports trust (Counties Manukau Sport) or by the District Health Board (Waikato, Northland), responsible for providing or funding the provision of health services in a given district. In total, Project Energize is being delivered to 53,375 children in 302 schools, or 15 % of all schools in NZ.

**Table 2:** Number of schools and children receiving the project Energize health service (2014)

Funded by	Delivered by	Primary schools (n)	Decile 1–3 (%)	Children (n)	Māori (%)
Waikato DHB	Sport Waikato	242	32	42,000	36
Counties Manukau Sport	Counties Manukau Sport	15	33	4850	25
Northland DHB	Sport Northland	45	100	6525	71
Energize total		302		53,375	42
NZ totals from ministry of education		1961	30		23
Energize as a % of the NZ total		15	22		

Deciles 1–3 are the most socially disadvantaged

As subsequent information collected was for the purpose of implementing a programme delivered through schools rather than for research purposes, approval by an ethics board was not required.

### Evaluation of the programme expanded to other settings

Randomised controlled trials are the cornerstone of proof of efficacious (more good than harm) outcomes, but practically it is not ideal/possible to have children engaged as controls for long periods of time. Further, a continuing challenge [16] with a long-lived programme, is to measure the ongoing effectiveness in the target population. In NZ, parental consent is required to measure children, which is expensive both in time and resources, with the reach of the measures being biased towards those who consent to be measured. It is part of the nature of real world implementation that information collected should be of immediate relevance to the schools themselves.

A primary measure of the effectiveness of Project Energize in the new primary schools is the measurement, before and 2 years after the intervention has been in place, of how fast children can run 550 m. The data are then compared to gender and age-specific run centiles developed from Waikato region data collected in the 2011 evaluation [17, 18]. Run-time is straightforward to measure and in New Zealand does not require parental consent if data are aggregated and de-identified. Similarly, additional aggregated and de-identified data on food and drink habits and length of time spent undertaking physical education, sport and fitness in the school setting data are collected from new clusters of participating schools via the distribution of standard parent and teacher questionnaires. This information is then returned to the provider and funder at baseline and 2 year follow-up. As well as informing whether the programme should continue to be supported, this information provides immediate feedback to the Energizers and schools as to where to focus their adaptable action plans e.g., more PA for older classes.

Unlike the original randomized, controlled trial, the review of implementation outcomes [19] is another key component of the continued evaluation of the Energize initiative. Since the inception of the initiative, meticulous documentation, in real time, has enabled feedback to be delivered directly to the funding body (Waikato District Health Board) about the progress of the programme. Each week the Energizers document activities they have undertaken with each school that they work with on to a database. Every term (10 weeks), these reports are collated and sent to the Waikato District Health Board. The report is quantitative and records over 10 weeks the number of schools, classes and children participating in PA and healthy eating activities (Table 3). The activities that are recorded are dependent on each school’s individualized action plan. Energizers cannot address every goal with each of their 8–12 schools each term, nor do the schools want to address every goal each term. However, all recorded activities can be linked to one or more of the healthy eating and physical activity goals (Table 1). The report also includes success stories and photographs that the Energizers have assembled about their schools, and these positive success stories are shared with the schools. Documentation additionally involves continuous formative evaluation of what worked and what didn’t work during the delivery of the intervention. Such evaluations revealed, for example, that translating resources into ‘Te Reo,’ the Maori language, and starting small with no pressure are approaches that ‘work’; that telling schools what they should be doing does not work; and that larger schools take longer to implement the programme. Such pragmatic implementation and accountability allows for ongoing modifications to the programme and tailoring for each school, allowing the initiative to continue to strive towards the goal of achieving the improve the overall health through increased physical activity and healthier eating and thus reduce the rate of weight gain, in the most efficient way [20].

**Table 3:** Example of a report of frequency of physical activity and healthy eating activities by number of schools, classes and children in 242 Energize schools over 10 weeks by 27 energizers

Healthy eating (HE goals 1–9)		1	2	3	4	5	6	7	8	9
# of schools that …										
Published the Energize nuggets?	198									x
Made healthy changes to their lunch order/canteen?	15						x			
Changed their fundraising from unhealthy food	13									x
Signed up for a heart start award this term?	11								x	
Were awarded a heart start award this term?	0								x	
# of classes that …										
Participated in a sugary drinks session?	22	x	x	x						
Participated in a takeaways session?	18					x	x			
Participated in a breakfast session?	16							x		
Participated in a Pro Joe’s lunchbox session?	14					x				
Participated in a four food groups session?	13	x	x	x	x	x	x			
Participated in a supermarket session?	2					x				
Participated in a food for fuel session?	0					x	x			
Participated in other nutrition sessions?	9			x	x	x				
# of parents who attended a healthy eating session	44					x				

Physical activity (PA goals 1–7)		1	2	3	4	5	6	7		
# of schools that …										
Had daily ex sessions modelled?	59				x	x	x			
Had SportsForce workshops?	49						x			
Had sports modelled without SportsForce?	95						x			
Did some kind of leadership training?	104					x		x		
Participated in cycling activities?	45						x			
Did fundamental movement skills testing?	12						x			
Did fitness testing?	4							x		
# of classes that …										
Did fundamental movement skills testing?	111						x			
Did fitness testing?	17							x		
# of sessions modeling …										
Daily ex	366				x	x	x			
Non-SportsForce	478						x			
Other activity sessions	290					x	x			
Fundamental movement skills testing	39						x			
# of students who participated in cycling activities	3467						x			
# of teachers who attended a professional development session	242					x		x		

## Expansion to other populations

### Under-five age group

In addition to expansion to a wider geographical area, Project Energize is being adapted and delivered to preschools in four socially disadvantaged areas in the Waikato region. The under 5’s intervention follows the same protocol as the original programme but has only six goals; “more active play every day”, “milk and water as the best choice”. “less sweet drinks”, “daily fruit and vegetables”, “less energy dense snacks” and “less screen time”. Both programmes have the mantra “eat healthy, be active, have fun” which is written on the side of the cars used. In the under 5 Energize programme, four Energizers look after 30 preschools each. This expansion of Project Energize to a pre-school setting is funded by the Ministry of Health and like the original programme, is being delivered by Sport Waikato and evaluated by Auckland University of Technology.

### Application in Cork, Ireland

Project Energize has also been adapted to be piloted in seven intervention (N = 2089) and three control (N = 1115) primary schools in Cork, Ireland. The Irish Energize programme, titled ‘Project Spraoi’ (pronounced Spree), follows the same protocol and adopts the same goals as the NZ intervention. Only minor adjustments have been made to the programme in order to make it appropriate for an Irish setting, such as adapting activities for play on concrete surfaces instead of grass. Two experienced NZ Energizers visited Ireland prior to the project commencement (August 2013) to provide training and support, and Sport Waikato continues to provide full logistical support for Project Spraoi.

## Discussion

Project Energize has proven to be an effective as well as efficacious school-based programme to combat obesity in children. A unique aspect has been success in maintaining the programme, which celebrated its 10-year anniversary in 2015. With respect to understanding and effecting maintenance and sustainability of a public health intervention programme like Energize, four aspects set it apart from other public health programmes. First and foremost is its well-trained, stable and knowledgeable workforce [21]. The selection of people for the Energizer role is essential to the ability of the programme to penetrate schools and work with them [22]. Energizers have diverse tertiary qualifications and experience in dietetics, sport, fundamental movement skills, physical education and teaching. The Energizer should live near or within the geographical area that they serve, so that they are seen as part of the community. Moreover, Energizers have secure employment as the programme is a standard budget line for the District Health Board and renewal is part of the annual cycle; the programme is not dependent on the good will of volunteers or local community leaders—an aspect that lends a great deal of stability to the programme.

Secondly, the programme is uniquely sensitive to inequity and to cultural differences. By design, over one-third of the children reached by Energize and 9 of the 27 Energizers are Māori. All resources are translated into Te Reo; methods of delivery consider Māori culture; and Māori games and activities are featured as an integral part of the intervention.

A third feature of the Energize programme that contributes to its continued effectiveness is its accountability. Research and evaluation has been an ongoing characteristic of the quality assurance for the programme, including measures of efficacy as well as of implementation adherence. Immediate feedback from schools to Energizers to Sport Waikato also allow for critical adjustments to programme activities to meet each individual school’s needs and enhance its participation.

Finally, successful maintenance of the program is enhanced by community and food environments that support what is modelled in schools—for example, in New Zealand, Fonterra’s introduction of free milk to all primary schools that opt-in (https://www.fonterramilkforschools.com/). As another example, in the Waikato region, a few supermarkets also work with Energizers on a weekly “meal deal,” where the healthy and simple recipes displayed at the front of the store use ingredients that are currently on promotion. Another supermarket chain offers ‘food for thought’ (http://www.foodforthought.co.nz) that assists year 5 and 6 primary school students make healthier food and lifestyle choices by providing class resources, nutritionist support and hosting supermarket visits.

The next step for each of the Energize programmes is to further research and document the process of implementation so that similar and future interventions may be informed by this experience. The challenge for Energize is to find ways to be sustainable, to reach the wider community and to work with others to create supportive environments.

## Conclusions

Project Energize, a cost-effective, community-based, through-school health promotion intervention, has been successfully running in NZ since 2005. It is inclusive of all children and currently involves over 300 primary schools in and beyond the Waikato region. Although other root causes, such as social deprivation and the quality of the commercial food supply, need to be addressed to really have a meaningful effect, it is hoped that the continued rollout of the programme will provide, in the long term, data to show that the prevalence of childhood overweight and obesity is not increasing, particularly in the Waikato region. In the meantime the documentation of the process of implementation and dissemination of Energize will continue to be evaluated, applied and shared with others.
